# Evaluating Mechanisms of IDH1 Regulation through Site-Specific Acetylation Mimics

**DOI:** 10.3390/biom11050740

**Published:** 2021-05-16

**Authors:** Joi Weeks, Alexandra I. Strom, Vinnie Widjaja, Sati Alexander, Dahra K. Pucher, Christal D. Sohl

**Affiliations:** 1 Department of Chemistry and Biochemistry, San Diego State University, San Diego, CA 92182, USA; joi.weeks@gmail.com (J.W.); alexandrastrom17@gmail.com (A.I.S.); vinniewidjaja28@gmail.com (V.W.); satiamrita@gmail.com (S.A.); pucher@usc.edu (D.K.P.); 2 Department of Biological Sciences, University of Southern California, Los Angeles, CA 90089, USA

**Keywords:** acetylation, IDH1, kinetics, post-translational modification

## Abstract

Isocitrate dehydrogenase (IDH1) catalyzes the reversible NADP^+^-dependent oxidation of isocitrate to α-ketoglutarate (αKG). IDH1 mutations, primarily R132H, drive > 80% of low-grade gliomas and secondary glioblastomas and facilitate the NADPH-dependent reduction of αKG to the oncometabolite D-2-hydroxyglutarate (D2HG). While the biochemical features of human WT and mutant IDH1 catalysis have been well-established, considerably less is known about mechanisms of regulation. Proteomics studies have identified lysine acetylation in WT IDH1, indicating post-translational regulation. Here, we generated lysine to glutamine acetylation mimic mutants in IDH1 to evaluate the effects on activity. We show that mimicking lysine acetylation decreased the catalytic efficiency of WT IDH1, with less severe catalytic consequences for R132H IDH1.

## 1. Introduction

Isocitrate dehydrogenase 1 (IDH1) is a homodimeric enzyme that catalyzes the NADP^+^-dependent conversion of isocitrate to α-ketoglutarate (αKG) in the cytoplasm and peroxisomes. IDH1 point mutations, most commonly R132H, drive > 80% of lower grade gliomas and secondary glioblastomas by catalyzing the neomorphic production of the oncometabolite D-2-hydroxyglutarate (D2HG) from αKG [1,2,3]. D2HG can inhibit αKG-dependent enzymes like DNA and histone demethylases, leading to genome hypermethylation and cell de-differentiation [4,5]. Mutant IDH1 is a therapeutic target, with a selective inhibitor currently in the clinic [6]. Increased wild type (WT) IDH1 activity has also been implicated in cancer [7,8,9].

Although IDH1 has been characterized biochemically [7,10,11,12], less is known about how human IDH1 is regulated. While IDH1 is conserved across many species, mechanisms of regulation appear to be divergent [13,14,15]. For example, yeast IDH1 is allosterically regulated by AMP, while human IDH1 appears to lack an AMP binding site [14]. In bacteria, IDH phosphorylation at residue S113 leads to inhibition [13,14,15]. While this residue is structurally conserved in human IDH1 (S94), it does not appear to be phosphorylated in humans. Instead, D279 hydrogen bonds with S94 to mimic the structural consequences of serine phosphorylation [13,14,16]. Phosphorylation may still prove to be important for IDH1 regulation; it has been reported that phosphorylation at Y42 and Y391 is important for cofactor binding and IDH1 dimerization, respectively [17].

Lysine acetylation is a common regulatory strategy to alter protein structure and function. Acetylation is catalyzed by lysine acetyltransferases, though there is evidence that acetylation can also occur non-enzymatically [18]. While protein acetylation is commonly associated with histone regulation, many cytosolic [19,20,21,22,23] and metabolic [23,24,25] enzymes are also regulated by this post-translational modification (PTM). Importantly, mitochondrial IDH2, which has high sequence and structural homology to IDH1 and catalyzes the identical reaction, is regulated via acetylation [26,27]; K413 acetylation results in a ~44-fold decrease in activity, with the deacetylase SIRT3 able restore this activity [26]. The catalytic consequences of K413 and K256 acetylation in IDH2 were evaluated using lysine to glutamine (K-to-Q) mutants in vitro, which mimic acetylated lysine residues, and both mutants showed inhibited catalysis [27].

Proteomics studies have indicated that several lysine residues in IDH1 are acetylated, including K81, K224, and K321 [28,29,30]. These residues are located near the active site of IDH1, and K224 and K321 are conserved in IDH2, but K81 is not [31]. Recently, K224 acetylation in IDH1 was investigated in colon cancer and was shown to have an inhibitory role [32], though the consequences of K81 and K321 acetylation in IDH1 have yet to be explored. Here, we investigate the effects of acetylation on IDH1 activity by measuring steady-state kinetic parameters of acetylation mimics in WT and R132H IDH1. We show that mimicking acetylation primarily leads to a decrease in IDH1 activity, particularly in the WT background. Overall, our work supports the growing evidence that IDH1 is regulated by lysine acetylation.

## 2. Materials and Methods

### 2.1. Reagents

Tris–hydrochloride, sodium chloride, magnesium chloride hexahydrate, and α-ketoglutarate (αKG, sodium salt) were purchased from Fisher (USA). The pH of αKG stocks was adjusted to 7.0 before use. *DL*-isocitric acid trisodium salt hydrate was purchased from MP Biomedicals (USA). NADPH (tetrasodium salt) and NADP^+^ (disodium salt) were purchased from Calbiochem (USA). Dimethyl sulfoxide (DMSO) was purchased from Sigma–Aldrich (USA). Bovine serum albumin (BSA) was purchased from SeraCare Lifescience (USA).

### 2.2. Construct Preparation

All IDH1 constructs were in a pET-28b vector containing an *N*-terminal hexahistidine tag. Site-directed mutagenesis (Kapa Biosciences, Wilmington, MA, USA) was used to generate K81Q (forward primer, 5′-GCCACC ATTACACCGGATGAACAGCGTGTGGAAGAATTTAAAC-3′, reverse primer, 5′-GTTTAAATTCTTCCACACGCTGTTCATCCGGTGTAATGGTGGC-3′); K224Q (forward primer, 5′-CCATTCTGAAAAAATACGATGGTCGCTTTCAGGATATTTTTCAAGAAATTT ACGATAAAC-3′, reverse primer, 5′-GTTTATCGTAAATTTCTTGAAAAATATCCTGAAAGCGACCATCGTATTTTTTCAGAATGG -3′); K321Q (forward primer, 5′-CGTCATTATCGTATGTATCAGCAGGGTCAAGAAACCAGCACCAAT-3′, reverse primer, 5′-ATTGGTGCTGGTTTCTTGACCCTGCTGATACATACGATAATGACG-3′); and D79L (forward primer, 5′- gttaaatgcgccaccattacaccgctggaaaaacgtgtggaagaatttaaa-3′, reverse primer, 5′-tttaaattcttccacacgtttttccagcggtgtaatggtggcgcatttaac-3′). All constructs were confirmed by sequencing.

### 2.3. Kinetic Assays

All proteins were expressed and purified using conditions published previously [3,33]. Purity (≥ 90%) was confirmed by SDS-PAGE, and IDH1 was flash frozen in liquid nitrogen and stored at 80 °C for no longer than 1.5 months. A diode array spectrophotometer was used (OLIS, Athens, GA, USA) for all enzymatic assays. Enzymatic concentrations were optimized for each protein to ensure optimal signal and linear range. For the conversion of isocitrate to αKG [3], the reaction was initiated by adding saturating concentrations of NADP^+^ (200 µM final concentration) and varying concentrations of isocitrate to limiting concentrations of IDH1 protein (100 nM for K81Q, K224Q, and K321Q IDH1, and 21 µM for D79L IDH1) in IDH1 assay buffer (50 mM Tris at 4 °C, pH 7.5 at 37 °C, 150 mM NaCl, 10 mM MgCl_2_, 1 mM dithiothreitol). For the neomorphic conversion of αKG to D2HG [3], the reaction was initiated by adding saturating concentrations of NADPH (200 µM final concentration) and varying concentrations of αKG to limiting concentrations of the R132H IDH1 double mutants (R132H/K81Q, R132H/K224Q, and R132H/K321Q IDH1, 500 nM) in assay buffer. The change in absorbance due to NADPH formation or depletion was monitored at 340 nm. For both reactions, the slopes of the linear range of the incubations were calculated and converted to nM NADPH using the molar extinction coefficient for NADPH of 6.22 cm^−1^ mM^−1^ to obtain *k*_obs_ (i.e., nM NADPH/nM enzyme s^−1^). Results were fit to hyperbolic plots in GraphPad Prism (USA) to estimate *k*_cat_ and *K*_m_ mean values ± standard error (S.E.) Assays were performed with at least two protein preparations.

### 2.4. Structural Modeling

K81Q, K224Q, and K321Q IDH1 were modeled into a structure of WT IDH1 in complex with isocitrate and NADP^+^ (PDB 1T0L [14]), and R132H IDH1 in complex with αKG and NADP^+^ (PDB 4KZO [34]) using PyMOL [35,36]. eLBOW in the Phenix software suite [37] was used for ligand restraint generation and optimization of cif files, and mutations were made directly in the PDB files using Coot [38]. Geometry Minimization in the Phenix software suite [37] was performed to regularize geometries (bond lengths, nonbonded distances, bond angles, dihedral angles, chirality, planarity, and parallelity) of the IDH1 mutant models in complex with the ligands, with 500 maximum iterations and 5 macro cycles.

## 3. Results

### 3.1. Glutamine Mutation Results in Decreased Kinetic Activity

Previously reported mass spectrometry data sets from human, mouse and rat samples showed lysine positions including K81, K87, K115, K224, K233, K243, and K321 were acetylated in WT IDH1 [28,29,30]. K81 and K224 were found to be acetylated in each of these datasets [28,29,30], with K321 found to be acetylated in two of the three data sets [28,30]. Further, one of these datasets identified K81, K224, and K321 as the only residues acetylated in IDH1 [30]. Together, this guided our selection of residues to probe. However, we note that in the datasets finding the additional acetylated residues in IDH1, a few features may explain the discrepancies. In [29], both peptides containing K87 and K115 are very short (seven residues each), which can be challenging to detect and/or assign, particularly when studying PTMs. As the peptides containing acetylated K233 and K243 [29] are longer, there must be other reasons for the discrepancy such as different stringencies in filtering strategies. We found that K81 in IDH1 was not conserved in IDH2, but K87, K115, K224, K233, K243, and K321 were [39]. It has previously been shown that acetylation of K256 and K413 in IDH2 inhibits activity [26,27], and we noted that K256 IDH2 (K217 in IDH1) and K413 IDH2 (K374 in IDH1) were conserved in IDH1. However, there was no evidence of acetylation of these residues in IDH1 in the proteomic data [28,29,30]. Lysine acetylation in or near the active site can affect substrate binding [40,41,42] or cause conformational changes that affect rates of catalysis [27,41,43]. Thus, acetylated lysine residues near the IDH1 active site (K81, K224, and K321) were prioritized for study [28,30]. Of note, K224 IDH1 is structurally proximal to K256 in IDH2 (corresponding to K217 in IDH1), which upon acetylation inhibits IDH2 catalysis [27]. Thus, we expected that mimicking acetylation at K224 in IDH1 would also decrease catalytic activity.

**Figure 1:**
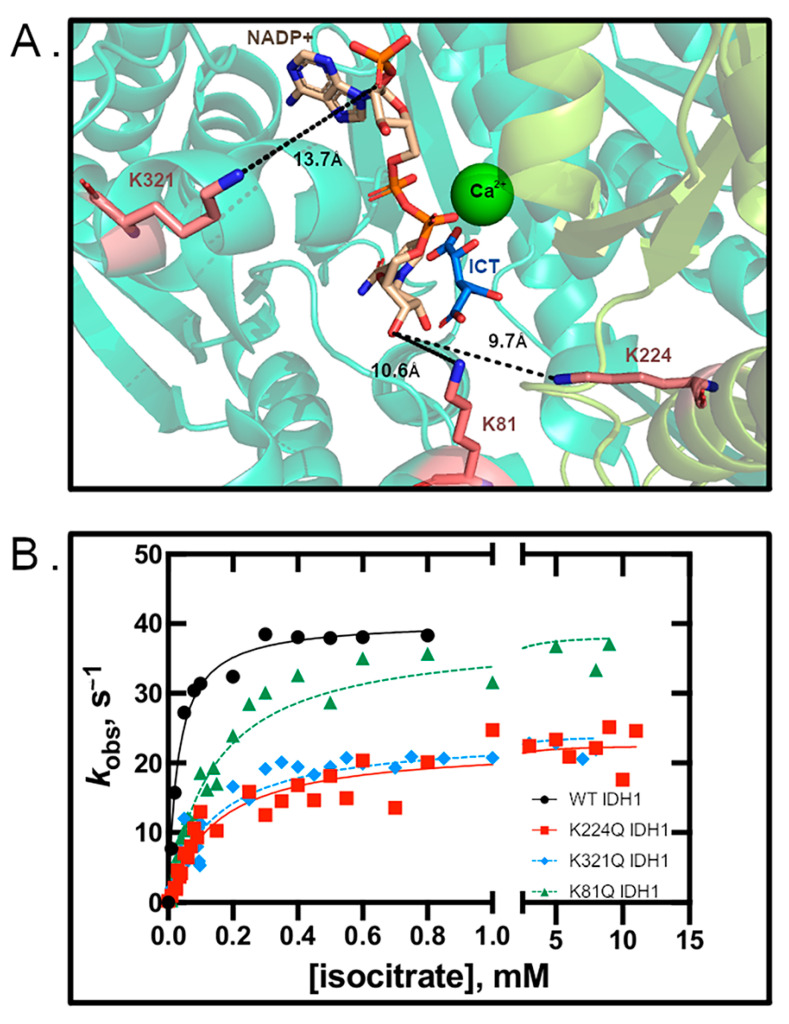
Mimicking acetylation inhibits WT IDH1 activity. (**A**) The active site of IDH1 with lysine residues shown in coral, and their distances from NADP^+^ are shown in Å (PDB 1T09 [14]). (**B**) Michaelis–Menten plots of WT and mutant IDH1.

Lysine acetylation mimics were used to probe the consequences of acetylation on WT and R132H IDH1 activity. Acetylation mimics K81Q, K224Q, and K321Q IDH1 were created in WT and R132H IDH1 backgrounds. This strategy has been successful for elucidating the catalytic and structural consequences of lysine acetylation for proteins such as IDH2, malate dehydrogenase, and histone H3 [18,27,44,45,46]. Steady-state kinetics experiments [3,13,27] probing the rates of αKG production were measured, and we found that K81Q IDH1 had a similar *k*_cat_ to WT IDH1, while K224Q and K321Q IDH1 had *k*_cat_ values reduced by >80% (Figure 1 and Table 1). All acetylation mimics had similar *K*_m_ values, which were 5-fold higher than that observed for WT IDH1. Thus, the catalytic efficiency of the IDH1 mimics decreased ~5-fold for K81Q IDH1 and ~8-fold for both K224Q and K321Q IDH1, driven primarily through a decrease in *k*_cat_. These results indicate that mimicking acetylation at residues K81, K224, and K321 in WT IDH1 leads to decreased αKG production efficiency.

### 3.2. R132H IDH1 Acetylation Mimics Have Minor Kinetic Effects

**Figure 2:**
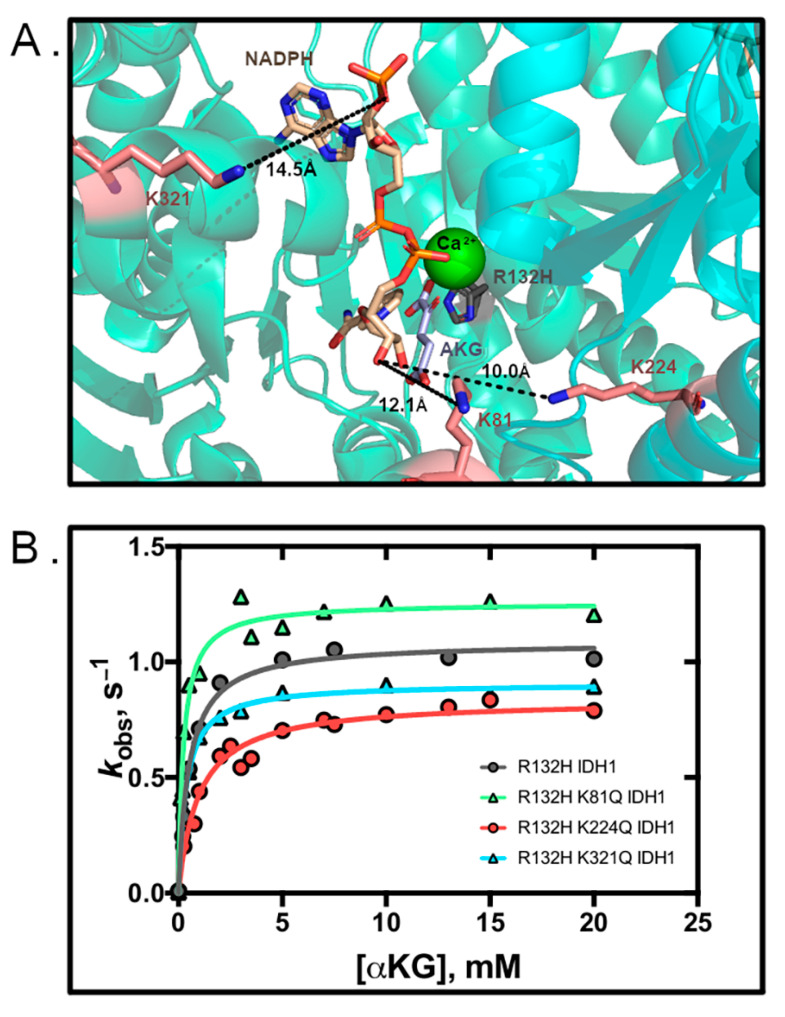
Mimicking acetylation has only modest effects on R132H activity. (**A**) The active site of R132H IDH1 with lysine residues identified in red and distances from NADPH are shown in Å (PDB 4KZO [34]). (**B**) Michaelis–Menten plots of IDH1 mutants.

We also evaluated whether mimicking lysine acetylation in the R132H IDH1 background similarly affected catalysis. R132H IDH1 is the most common point mutant found in IDH1 tumors, and this mutant catalyzes the neomorphic reaction of D2HG production from αKG [1]. Double mutants (R132H/K81Q, R132H/K224Q, and R132H/K321Q IDH1) were assessed for catalytic efficiency of D2HG production. Only modest changes in *k*_cat_ and *K*_m_ values were observed in the double mutants as compared to R132H IDH1 (Figure 2 and Table 2). Mutations at residues K81Q/R132H and K224Q/R132H IDH1 had the largest impact, with a 2-fold increase (K81Q/R132H IDH1) or decrease (K224Q/R132H IDH1) in catalytic efficiency that was driven primarily through an increase in *K*_m_. We conclude that mimicking lysine acetylation in R132H IDH1 can only have modest yet varied consequences on catalysis.

### 3.3. Residue D79 Plays an Important Role in WT IDH1 Activity

**Figure 3:**
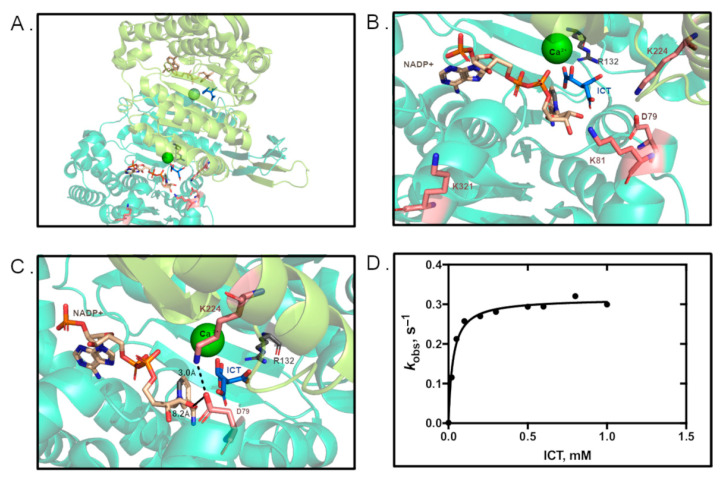
The D79L mutation ablates IDH1 activity. (**A**) IDH1 WT (PDB 1T09 [14]). (**B**) Active site of IDH1 in the same orientation shown in A, with mutated residues and substrates indicated. (**C**) IDH1 active site, highlighting D79 and its distance to K224. (**D**) Michaelis–Menten plot of D79L IDH1.

Of the three lysine residues studied, only K224 had close proximity to other residues to engage directly in noncovalent interactions as observed in crystal structures. As shown in Figure 3, K224 is ~3 Å away from D79′ [14]. The proximity and orientation of these two residues suggest that these domains could be stabilized through a salt bridge interaction, and that this interaction could be disrupted upon acetylation of K224. Residues upstream from D79 help form part of the NADP(H) binding pocket [14], so stabilization of these domains could impact catalysis, though D79 itself is fairly distant from the active site (8.2 Å from NADP^+^). To probe the importance of interactions between K224 and D79, we designed a D79 mutant that would destroy the potential for hydrogen bonding or salt bridge interactions with K224, but otherwise mimic the structure of an aspartic acid residue (D79L). Using steady-state kinetics, we determined that the *k*_cat_ of D79L IDH1 drastically decreased by > 99% to generate a catalytic efficiency that is 130-fold lower (Figure 3, Table 1). Detection of any activity by D79L IDH1 required a 21-fold increase in concentration of the enzyme. To our knowledge, D79 has not previously been identified in playing a role in IDH1 catalysis.

### 3.4. Structural Modeling of IDH1 Mutations

**Figure 4:**
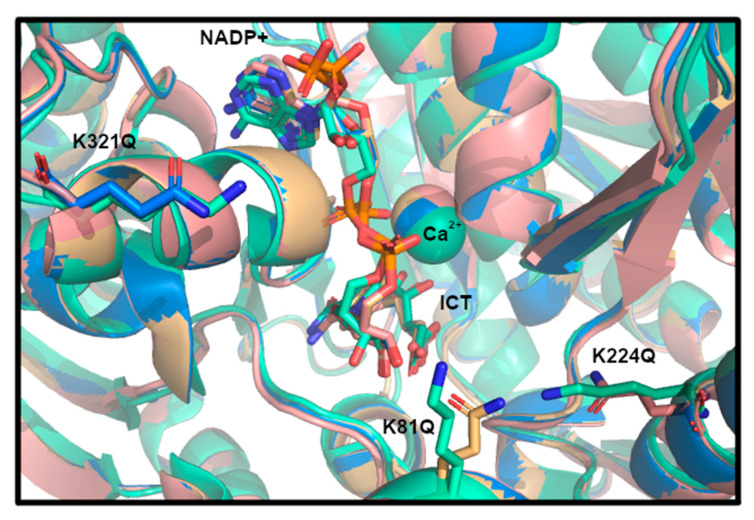
Structural modeling of acetylation mimics in IDH1. WT IDH1 crystal structure complexed with isocitrate (ICT), NADP^+^, and Ca^2+^ in the active site (PDB 1T0L [14]) was used for modeling. WT IDH1 is displayed in teal, K81Q IDH1 in orange, K224Q IDH1 in salmon, and K321Q IDH1 in blue.

To predict if any major structural changes might result from K81Q, K224Q, and K321Q IDH1 mutation, the K-to-Q mutants were modeled in previously solved crystal structures of WT IDH1 (PDB 1T0L [14]) using the geometry minimized package available in the Phenix software suite [37]. Alignment of the mutant models to the original WT IDH1 structures were performed in PyMOL to identify minimal global changes at the K-to-Q mutation positions. No major changes to the structure of WT IDH1 were noted, though crystallographic characterization is needed to confirm this observation (Figure 4).

## 4. Discussion

Here, we report that mimicking acetylation of residues K81, K224, and K321 in IDH1 results in a decrease in catalytic activity in WT IDH1, and more varied and minor effects on R132H IDH1 that may not be notable enough to have physiological ramifications. An interesting future direction is to determine if simultaneous acetylation of lysine residues have an additive or synergistic effect. Recently, K224 in IDH1 was shown to be deacetylated by SIRT2 to inhibit colorectal cancer and liver metastases [32]. K224R IDH1, a deacetylation mimic, and K224Q IDH1 were stably expressed in 293T cells, resulting in inhibited IDH1 activity [32]. Our data support their kinetic findings [32]. They also evaluated the effects of K81 and K321 IDH2 by expressing deacetylation mimics in 293T cells, but the acetylation levels at these positions as assessed by Western blot were unchanged compared to WT IDH1 [32], and thus no further characterization was undertaken. While acetylation mimics are valuable for understanding the potential kinetic consequences of lysine acetylation, one limitation is that they cannot provide mechanistic information on the consequences of the PTM. Instead, systems biology methods in which acetylated lysine residues are incorporated during translation using N^e^-acetyllysyl-tRNA synthetases/tRNAs are the gold standard to identify the global consequences of protein acetylation in cells [47] and represent an important future direction in understanding the role of acetylation in regulating IDH1.

We also undertook preliminary efforts to probe the structural consequences of lysine acetylation. Of the three lysine residues explored here, K224 IDH1 appeared to have the greatest potential to noncovalently interact with a nearby residue (D79) via a salt bridge or hydrogen bonding. Mutation of D79 (D79L IDH1) resulted in nearly full ablation of WT IDH1 activity, revealing the importance of this residue in catalysis. Future work includes using structural methods to observe the consequences of the K224Q mutation, particularly in context of interactions with D79.

Overall, using steady-state kinetics, we elucidated the catalytic consequences of lysine residues reported to be acetylated in IDH1 [28,29,30]. Kinetic parameters for a series of K-to-Q mimics, a valuable strategy for understanding the consequences of acetylation in vitro [18,27,41,44,46], were reported in both WT and R132H backgrounds. We show that mimicking acetylation of three lysine residues (K81, K224, and K321) in IDH1 led to inhibition in WT IDH1 and variably altered activity in R132H IDH1, suggesting that IDH1 acetylation may have a regulatory role, though in vivo cellular experiments are still required. Acetylation of K224 IDH1 may affect interaction with D79, a non-active site residue that we show here is important for catalysis. Overall, this study provides a foundation for determining mechanistic consequences of IDH1 acetylation as a means of regulation.

## Figures and Tables

**Table 1:** Steady-state kinetics parameters for WT IDH1. The normal reaction, isocitrate conversion to αKG, was measured for WT IDH1 and acetylation mimic mutants and the S.E. was determined by deviation from the hyperbolic fits. At least two protein preparations were used when measuring *k*_obs_ rates.

IDH1	*k*_cat_, s^−1^	*K*_m_, mM	*k*_cat_/*K*_m_, µM^−1^ s^−1^
WT	40.4 ± 0.8	0.03001 ± 0.0008	1.3 ± 0.1
K81Q	38 ± 1	0.14 ± 0.02	0.27 ± 0.04
K224Q	23.0 ± 0.8	0.14 ± 0.02	0.16 ± 0.02
K321Q	24 ± 1	0.14 ± 0.02	0.17 ± 0.02
D79L	0.315 ± 0.006	0.028 ± 0.003	0.010 ± 0.001

**Table 2:** Steady-state kinetics parameters for mutant IDH1. The neomorphic reaction, αKG conversion to D2HG, was measured for R132H IDH1 and acetylation mimic mutants in the R132H IDH1 background. The S.E. was determined by deviation from the hyperbolic fits, and at least two protein preparations were used when measuring *k*_obs_ rates.

IDH1	*k*_cat_, s^−1^	*K*_m_, mM	*k*_cat_/*K*_m_, µM^−1^ s^−1^
R132H	1.09 ± 0.02	0.51 ± 0.04	0.0021 ± 0.0002
R132H/K81Q	1.26 ± 0.03	0.24 ± 0.03	0.0052 ± 0.0007
R132H/K224Q	0.84 ± 0.02	1.0 ± 0.1	0.0009 ± 0.0001
R132H/K321Q	0.91 ± 0.01	0.37 ± 0.02	0.0025 ± 0.0001
